# The Synthesis and Spectroscopic Characterization of Structural Changes in Hydrophobic Silica Aerogels upon Encapsulation of the LCC ICCG Enzyme

**DOI:** 10.3390/gels11020092

**Published:** 2025-01-25

**Authors:** Tatiana Alpízar-Rojas, Juan Diego Barboza-Carmona, Erik Butenschön, Guzel Musabirova, Erick Castellón, Jörg Matysik, Isaac F. Céspedes-Camacho

**Affiliations:** 1 School of Materials Science and Engineering, Instituto Tecnológico de Costa Rica, Cartago 30101, Costa Rica; tatialpizar15@gmail.com; 2 Environmental Protection Research Center (CIPA), Tecnológico de Costa Rica, Cartago 30101, Costa Rica; 3 Department of Industrial Engineering, Universitá degli Studi di Padova, Via Gradenigo, 6/a, 35131 Padova, Italy; juandiego.barbozacarmona@studenti.unipd.it; 4 Institute of Analytical Chemistry, Universität Leipzig, Linnéstr. 3, 04103 Leipzig, Germany; erik.butenschoen@uni-leipzig.de (E.B.); guzel.musabirova94@gmail.com (G.M.); 5 School of Chemistry, Universidad de Costa Rica, San José 11501, Costa Rica; erick.castellon@ucr.ac.cr; 6 School of Chemistry, Instituto Tecnológico de Costa Rica, Cartago 30101, Costa Rica

**Keywords:** silica aerogels, enzyme encapsulation, LCC ICCG, sol-gel synthesis

## Abstract

Silica aerogels are highly porous materials known for their low density and extensive surface area, making them ideal for applications in thermal insulation, catalysis, and environmental remediation. This study investigates the structural changes of functionalized hydrophobic silica aerogels used as carriers of the LCC ICCG enzyme. The aerogels were synthesized using the sol-gel method, with trimethylethoxysilane (TMES) as a functionalizing agent to enhance hydrophobicity. The enzyme-encapsulated aerogels were characterized using hyperpolarized ^129^Xe NMR, ^29^Si NMR, nitrogen sorption analysis, TEM, contact angle measurements, and FT-IR spectroscopy to evaluate their structural and chemical properties. The results confirmed successful encapsulation of the enzyme, as indicated by changes in the pore structure and network morphology. These findings demonstrate that functionalized silica aerogels can effectively support LCC ICCG immobilization, offering a promising approach for plastic degradation applications.

## 1. Introduction

Silica aerogels are highly porous solid materials known for their large surface area, low thermal conductivity, and reasonable chemical stability [1]. These properties have allowed them to find applications in various fields, including thermal and acoustic insulation, Cherenkov radiation detection, micrometeorite analysis, catalyst support, drug delivery, and the removal of contaminants from water [2,3,4,5,6]. Among these applications, a highly promising utilization of silica aerogels is enzyme entrapment, i.e., a process that involves incorporating enzymes within the aerogel matrix during synthesis to enhance their stability and reusability, resulting in efficient catalytic systems [6,7,8,9,10,11,12].

Immobilizing enzymes in silica aerogels offers numerous advantages, such as protecting them from adverse conditions and improving their thermal stability and reusability [13,14]. These advantages have increased interest in applications like enzymatic catalysis [15,16,17]. For instance, the use of silica aerogels as support for lipases and laccases has been shown to enable significant enzyme retention and maintain enzymatic activity even after multiple reuse cycles [18,19,20]. Additionally, the porous structure of silica aerogels can be adjusted to optimize enzyme loading and catalytic efficiency [21,22,23].

Platforms like cross-linked enzyme aggregates (CLEAs) and metal–organic frameworks (MOFs) have been widely explored for this purpose. While CLEAs offer high levels of catalytic activity and thermal stability, their dense structure often limits substrate diffusion. MOFs, on the other hand, provide high porosity and chemical versatility, but their application faces challenges in scalability, cost, and synthesis complexity for large-scale applications [22,23,24,25]. Silica aerogels have emerged as a compelling alternative due to their combination of low density, adjustable porosity, and hydrophobicity, which can support enzyme stability in aqueous conditions while facilitating substrate access [16]. Moreover, functionalization with agents like trimethylethoxysilane (TMES) allows precise customization of surface properties [26]. Despite these advantages, little research has explored the use of silica aerogels to immobilize enzymes designed for plastic degradation, such as the mutant of Leaf-Branch Compost 2x Cysteine and Glycine (LCC ICCG). Moreover, few studies have been done regarding the immobilization effects across silica carriers [13,23].

LCC ICCG is an optimized enzyme capable of achieving over 90% depolymerization of polyethylene terephthalate (PET) into monomers within hours under specific conditions [27,28,29]. This enzyme’s exceptional performance positions it as a key player in the biocatalytic recycling of PET waste, offering a sustainable and efficient alternative to traditional recycling methods [30,31,32,33,34,35,36]. While its free-form catalytic efficiency has been extensively studied, there is a significant gap in the understanding of how this enzyme can be integrated into advanced solid supports. Encapsulation in silica aerogels offers potential enhancement of the enzyme’s stability, reusability, and substrate accessibility, making the complex particularly effective for PET degradation [18,19,20,21,22,23]. Therefore, exploring the encapsulation of LCC ICCG in silica aerogels is a critical step toward enhancing its performance and enabling the development of efficient and sustainable PET recycling systems.

This study aims to evaluate potential structural changes (i.e., in the pore and framework structure, hydrophobicity, and specific surface area) in functionalized silica aerogels following the encapsulation, for the first time, of the enzyme LCC ICCG by using hyperpolarized (HP) ^129^Xe Nuclear Magnetic Resonance (NMR), ^29^Si and ^13^C NMR, nitrogen sorption analysis, contact angle measurements, Transmission Electron Microscopy (TEM), and Fourier Transform Infrared Spectroscopy (FT-IR).

## 2. Results and Discussion

Previous studies have examined the influence of various functionalizing agents on the surface area, hydrophobicity, and pore size properties of silica aerogels, including polyvinyl alcohol (PVA), polyethylene glycol (PEG), trimethylchlorosilane (TMCS), and trimethoxysilane (TMES). These studies demonstrate that the inclusion of TMES as a functionalizing agent is particularly effective in significantly enhancing the hydrophobicity of aerogels and enhancing their porous structure, maintaining a suitable balance between specific surface area and pore size [26]. Consequently, TMES has been identified as the most appropriate functionalizing agent for producing silica aerogels with hydrophobic properties. Two samples were synthesized and analyzed—a TMES-functionalized aerogel (A024: aerogel functionalized with TMES and an aging time of 24 h) and a TMES-functionalized aerogel that underwent the current enzyme encapsulation experiments (A0E24: aerogel with the enzyme).

**Figure 1:**
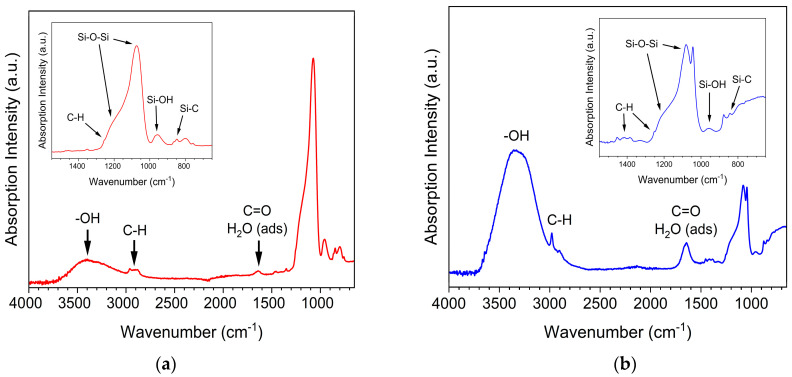
FT-IR spectra of the synthesized samples (**a**) A024 and (**b**) A0E24. Spectrum (**a**) shows Si–C and Si–O–Si vibrations, indicating surface modification with TMES. Spectrum (**b**) reveals bands for adsorbed water and C–O stretching, linked to enzyme encapsulation. Insets focus on the 1500–700 cm^−1^ region.

Figure 1 illustrates the FT-IR results of the synthesized samples. In sample A024 (Figure 1a), three peaks around 757, 847, and 867 cm^−1^ indicate the incorporation of methyl groups onto the silica surface through the formation of Si-C bonds, confirming surface modification with TMES. This modification replaces original silanol groups with hydrophobic methyl groups; consequently, the TMES inclusion significantly increased hydrophobicity, as evidenced by the contact angle measurements in Table 1 [26,36,37,38,39].

The incorporation of methyl groups enhanced the interaction with nonpolar environments. Mazaheri et al. [40] reported a similar enhancement in hydrophobicity upon introducing alkyl-functionalized ligands into silicate-phenolic networks. They highlight that these modifications are particularly effective in reducing surface free energy, an effect mirrored in our results for A024, suggesting a shared underlying mechanism of hydrophobic modification through surface functionalization. Moreover, the presence of strong Si-C bond signals in FT-IR further supports the structural integration of these groups, providing chemical stability to the modified silica aerogel.

Additionally, a peak at 800 cm^−1^ and a strong band at 1080 cm^−1^ correspond to ν_s_(Si-O-Si) (symmetric stretching) and ν_as_(Si-O-Si) (asymmetric stretching) vibrations, respectively, in the transverse optical (TO) mode which is related to the structural framework of the silica aerogels [40]. The peak at 1650 cm^−1^ is associated with δ(H-O-H) vibrations, indicating the presence of adsorbed water molecules within the aerogel matrix. A small peak in the 3000–3450 cm^−1^ range covers ν(Si-OH) and ν(H-O-H) vibrations, suggesting that the surface modification was not entirely complete, as some unreacted silanol groups remained on the silica surface and interacted with water molecules. The peak at 2964 cm^−1^ is attributed to ν_s_(C-H), also confirming surface modification with TMES [41].

In sample A0E24 (Figure 1b), a large peak in the region of 3400 cm^−1^ suggests the presence of a high amount of absorbed water molecules. While Jung et al. [42] attribute such behavior to limitations in reaction completeness, Mazaheri et al. [40] propose that specific network interactions and the structural flexibility of silicate frameworks can facilitate water retention. In the case of A0E24, both phenomena may contribute to the observed signals, as the enzyme encapsulation likely introduces additional hydrophilic sites. The notable peak around 1040 cm^−1^ indicates vibrational modes related to the C-O stretching in the enzyme, particularly associated with hydroxyl groups present in hemicelluloses, as observed in LCC ICCG enzyme structures. This signal is consistent with the presence of carbohydrate residues, such as trehalose [42,43].

In the upper left corner of the spectrum, a peak around 960 cm^−1^ indicates the presence of Si-OH groups. The relatively small peak suggests that most functional groups reacted during the condensation, leaving a small amount of unfunctionalized Si-OH groups on the aerogel’s surface [44,45,46,47].

Like sample A024, the C-H stretching vibrations can be seen at 2964 cm^−1^; however, an additional peak is observed at 1400 cm^−1^, corresponding to the same vibrations [36]. In the case of the A0E24 sample, the peak intensity is higher, likely due to the additional alkyl groups introduced through enzyme encapsulation. This observation is comparable to the results produced by Jung et al., where the introduction of hydrophobic alkyl groups led to a marked increase in hydrophobicity [42], as indicated by the contact angle measurements from 128.56° to 148.37° in Table 1.

Additionally, there is a peak at 1650 cm^−1^. This peak can be attributed to the Amide I and Amide II bands for C=O stretching in the enzyme’s peptide bonds, and near 1540 cm^−1^ for N-H bending and C-N stretching [48]. However, the broad shape of this peak also indicates H-O-H bending vibrations, which suggests a high amount of absorbed water molecules [27,48], which may mask the clearer detection of amino group signals in the enzyme.

Silicon environments are categorized into Q^n^ sites, where Q^4^ corresponds to fully condensed siloxane (Si-O-Si) structures, and Q^3^ and Q^2^ sites, which indicate partially condensed silanol (Si-OH) groups. The formation of Q^n^ and T^n^ sites results from the sol-gel process, which involves hydrolysis and condensation reactions. During condensation, silicon alkoxides (Si(OR)_4_) hydrolyze to form silanol groups (Si-OH), which then condense to form siloxane bonds (Si-O-Si), releasing water or alcohol as byproducts [49,50,51].

**Figure 2:**
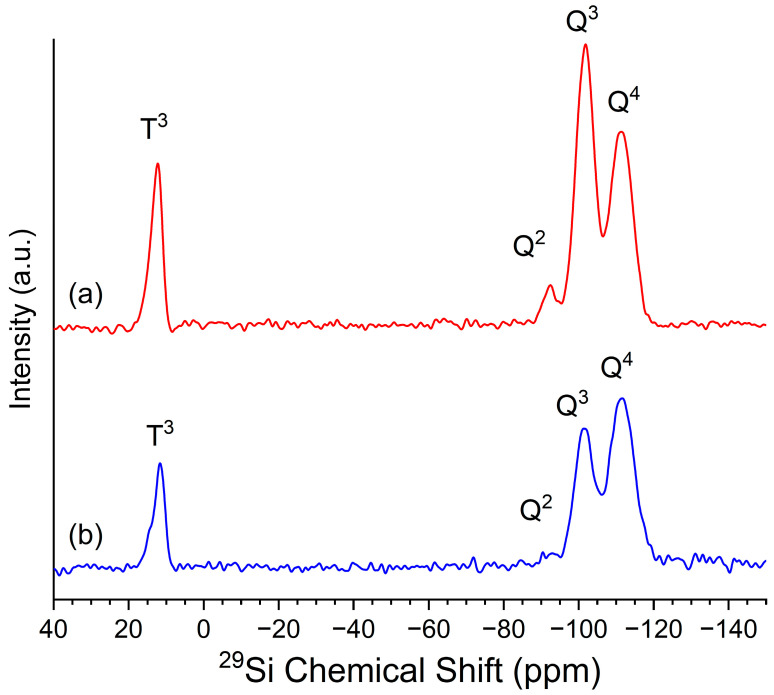
^29^Si NMR spectra of the synthesized samples (**a**) A024 and (**b**) A0E24. The peaks corresponding to different silicon environments are labeled as T^3^ (–O–Si(CH_3_)_3_), Q^2^ and Q^3^ (partially condensed silanol (Si-OH)), and Q^4^ (fully condensed siloxane (Si-O-Si)).

In sample A024, Figure 2a, the ^29^Si NMR spectrum reveals two signals at approximately −112.22 ppm and −101.23 ppm, corresponding to Q^4^ and Q^3^ sites, respectively. Q^4^ sites account for 39.68% of the total silicon atoms, indicating a condensed and crosslinked siloxane structure. Q^3^ sites represent 57.2% of the silicon network, suggesting the presence of residual silanol groups (Si-OH). A minor fraction of the Q^2^ sites (3.11%) is present at −92.25 ppm, indicating a small degree of incomplete condensation. The peak at 11.96 ppm corresponds to –O–Si(CH_3_)_3_, also called T^3^ groups, confirming successful surface modification with TMES as supported by the strong C-H stretching vibrations observed in the FT-IR spectra and the high contact angle results.

In the enzyme-encapsulated aerogel A0E24, Figure 2b, the ^29^Si NMR spectrum displays similar Q^3^ and Q^4^ chemical shift positions. However, variation in their integrals suggests that enzyme encapsulation influences the condensation of the material surface. The Q^4^ sites increased to 64.94%, indicating a much more densely crosslinked siloxane network than in the A024 sample. Both the Q^3^ and Q^2^ sites also show decreased integral ratios. This shift in Q^n^ distribution aligns with previous studies [51], which have shown that enzyme encapsulation within sol-gel matrices can facilitate the formation of additional Si-O-Si bonds by reducing the number of available silanol groups, resulting in a more compact and highly reticulated network.

**Figure 3:**
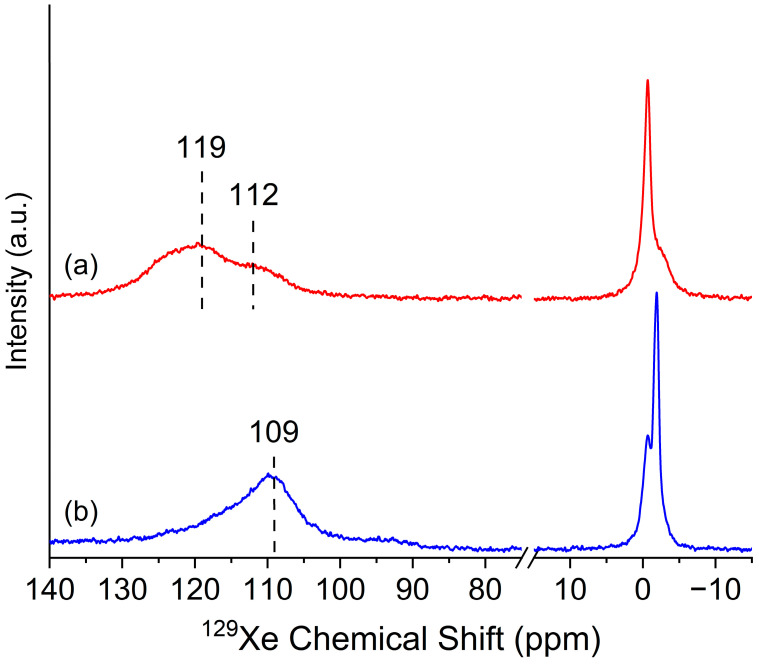
HP ^129^Xe NMR spectra of the synthesized samples (**a**) A024 and (**b**) A0E24. The peaks correspond to different pore environments, with chemical shifts reflecting pore size and structure. Higher chemical shifts indicate smaller pores.

The HP ^129^Xe NMR spectra of samples A024 and A0E24 are presented in Figure 3. The sample A024 (Figure 3a) exhibits two main signals at 119 and 112 ppm, which indicates Xe atoms trapped within the pores of the aerogel. These peaks suggest that the sample contains accessible pores at 213 K, a temperature at which exchange processes occur at a slower rate compared to the NMR timescale [52,53]. The peak position is influenced by the interactions between the Xe atoms and the pore surfaces. According to the relationship established by Telkki et al. [54], ^129^Xe chemical shift data can be translated into pore diameter information. The observed chemical shift corresponds to pore widths of approximately 6.2 and 6.9 nm [54,55]. Smaller pore sizes, or a reduced mean free path of xenon atoms within the sample, as proposed by Fraissard et al., result in decreased shielding effects and, thus, higher chemical shifts [26,55,56]. These findings are consistent with the characteristics of mesoporous materials, which typically exhibit pore diameters in the nanometer range [57].

For sample A0E24, Figure 3b, the HP ^129^Xe NMR spectra exhibit a similar pattern to A024, with one main signal at approximately 109 ppm, corresponding to a pore size of about 7.4 nm. The shift in the peak position relative to A024 suggests differences in the pore environment, potentially caused by changes in the surface interactions within the aerogel. Additional evidence from IR spectroscopy indicates the presence of a significant amount of water in the aerogel matrix with protein encapsulation. This observation suggests that water molecules may occupy pore surfaces, thereby reducing direct interactions between xenon atoms and the hydrophobic pore walls. Since xenon is hydrophobic, these changes likely weaken its interaction with the aerogel surface, contributing to the observed chemical shift at a higher field. These results align with findings from the literature that highlight the impact of hydrophilic environments on xenon’s NMR chemical shifts. Reduced xenon–wall interactions due to water coverage can alter the shielding effects, shifting the chemical shift to stronger fields [58]. Furthermore, these results align with ^29^Si NMR studies that demonstrate that enzyme encapsulation can facilitate additional Si-O-Si bond formation.

**Figure 4:**
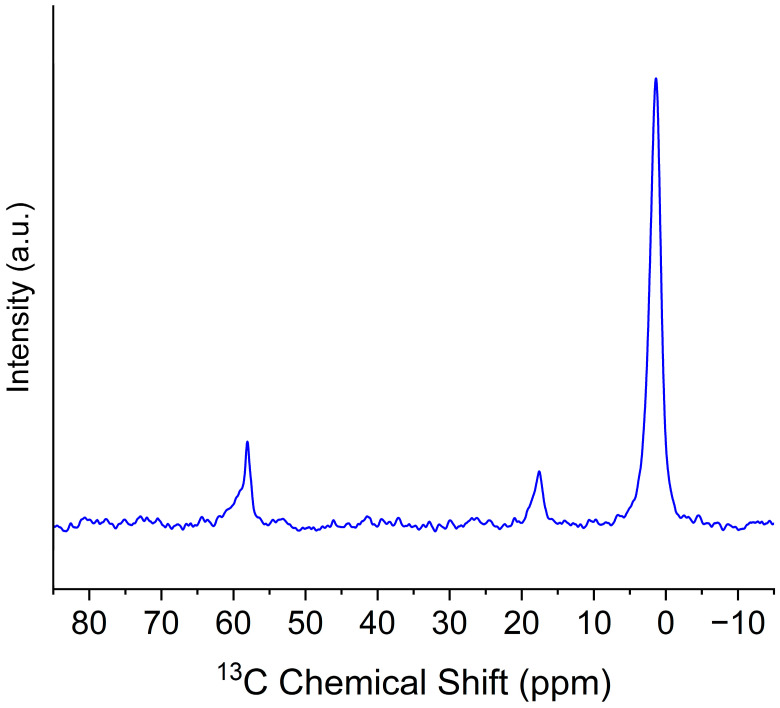
^13^C CP-MAS NMR spectra of the synthesized A0E24 sample. The peaks correspond to the carbon environments related to surface functionalization and the carbon network structure.

The ^13^C NMR spectrum of the A0E24 sample is presented in Figure 4. The peak at 1.45 ppm is assigned to the –Si–CH_3_ groups introduced by TMES. This peak is characteristic of surface methylation. The two signals at 58.11 ppm and 17.61 ppm are attributed to the CH_2_ and CH_3_ groups of the ethoxy group, respectively [45]. These chemical shifts are consistent with those reported in the literature for ethoxy-functionalized silica materials [45,55], confirming the successful incorporation of the –Si–OCH_2_CH_3_ groups onto the silica surface. The CH_2_ group, located adjacent to the oxygen, appears at the higher chemical shift (58.11 ppm), while the terminal CH_3_ group is observed at the lower chemical shift (17.61 ppm).

**Figure 5:**
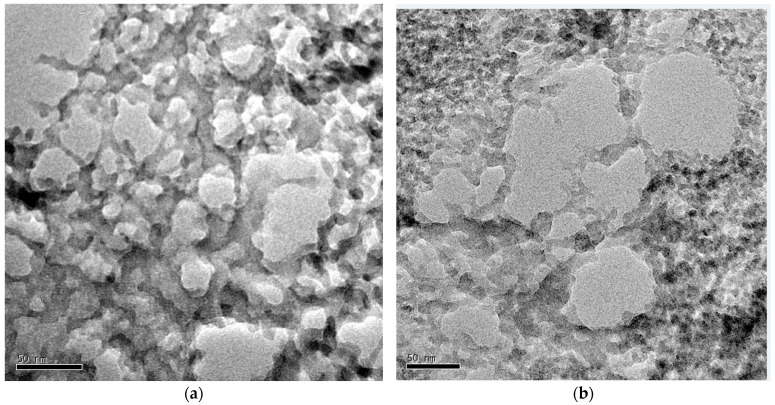
TEM micrographs of the synthesized aerogel samples: (**a**) A024, showing the characteristic porous structure; and (**b**) A0E24, showing the modified structure after LCC ICCG encapsulation.

The TEM micrographs of both A024 and A0E24, shown in Figure 5, are consistent with the typical interconnected particle structures seen in silica aerogels, reflecting the aging process in TMES [26,52]. In A024, the coarse structure indicates a well-formed porous network, with open spaces between the silica particles. This texture is characteristic of the robust aerogel framework resulting from extended aging, which promotes the development of a stable and interconnected network [59].

In A0E24, while the coarse structure is also observed, the micrographs reveal tighter particle aggregation, suggesting enzyme encapsulation’s impact. This denser arrangement of particles results in smaller pores and a more compact network, indicating that the encapsulation process influences the overall porosity and accessibility of the aerogel, as evidenced by the ^129^Xe NMR data. The enzyme encapsulation appears to reduce pore size, likely due to increased particle–particle interactions and structural rigidity. These differences in porous structure between A024 and A0E24 reflect the varying roles of surface modification and the incorporation of enzymes into the material.

**Figure 6:**
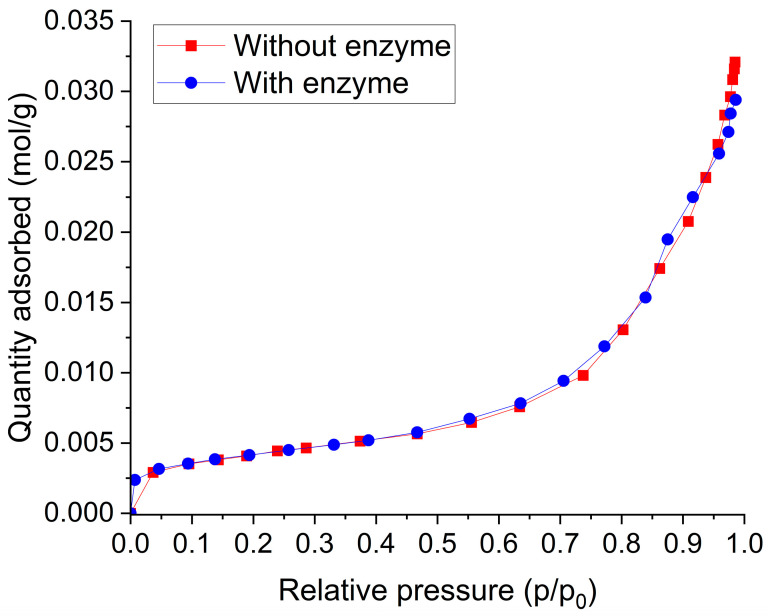
The adsorption isotherms of 77.15 K of aerogel with (circle, blue) and without (square, red) the enzyme. The isotherms provide information on the amount of gas adsorbed as a function of relative pressure, reflecting the available surface area and the interaction between the gas and the material.

The BET surface area and pore volume results (Table 1) show small differences between the functionalized aerogel and the enzyme-encapsulated aerogel. A024 has a higher surface area of 338 m^2^/g compared to 330 m^2^/g for A0E24, indicating that the enzyme encapsulation slightly reduces the accessible surface area. This alteration is not necessarily a result of the enzyme occupying the pores, but rather its modification of the pore distribution and accessibility, subtly limiting gas adsorption [60,61]. This behavior is supported by the adsorption isotherms (Figure 6) where A024 exhibits slightly higher nitrogen adsorption across the entire pressure range, particularly at higher relative pressures (p/p_0_ > 0.8). This suggests that enzyme encapsulation restricts pore accessibility and modifies the internal structure, correlating with the observed reduction in the BET surface area.

The HP ^129^Xe NMR results correlate with the decrease in the BET surface area and pore volume, indicating significant structural changes in A0E24 due to enzyme encapsulation. In A0E24, the pore size increases compared to A024, but the reduction in pore volume suggests that the total accessible pore space has decreased, limiting the number of pores available for xenon adsorption. The resulting shift in pore distribution impacts xenon–surface interactions, leading to stronger interactions, as reflected in the chemical shift in the NMR spectra.

Furthermore, the BET results, which show a lower surface area and reduced gas adsorption capacity in A0E24, support the idea that enzyme encapsulation leads to a denser, more compact structure. This structural compaction limits pore accessibility, reducing the material’s capacity to absorb gas. The shift in the HP Xe NMR spectra indicates redistribution of the available pore space, with Xe atoms interacting primarily with larger, enzyme-unblocked pores. The adsorption isotherms reinforce this interpretation, highlighting how the structural changes induced by enzyme encapsulation subtly alter gas–surface interactions and reduce adsorption capacity.

## 3. Conclusions

The results of this study demonstrate the potential of functionalized hydrophobic silica aerogels as effective carriers of the LCC ICCG enzyme. ^29^Si NMR showed a high degree of network condensation due to the integrals of the Q^3^ and Q^4^ signals. BET results highlighted a slight reduction in surface area and pore volume in enzyme-loaded samples, suggesting a more compact and less accessible pore structure. HP ^129^Xe NMR supported this conclusion, showing changes in pore size and stronger surface interactions. TEM images revealed a more compact, homogeneous morphology in enzyme-encapsulated aerogels, further corroborating the BET and NMR data.

FT-IR confirmed the successful modification of the silica surface, and the increased contact angle demonstrated enhanced hydrophobicity. Additionally, the ^13^C NMR results confirmed the presence of methyl groups from TMES. Moreover, the presence of the enzyme does not interfere with the surface modification of the aerogel, given the specific conditions shown in this study.

These findings suggest the potential use of the hydrophobic silica aerogels-LCC ICCG system for environmental applications involving PET degradation.

## 4. Materials and Methods

Silica gels were prepared from a reactive grade solution of sodium silicate (SS) with a molar ratio Na_2_O:SiO_2_ of 1:2.5 and SiO_2_ 26.5 wt.%. TMES was added as a surface modification agent, and a 0.5 mg/mL LCC ICCG enzyme solution in 50 mM sodium phosphate buffer with 200 mM NaCl (pH 7.4) was used for the enzyme entrapment. Nitric acid (HNO_3_) was used as a catalyst. Tetraethyl Orthosilicate (TEOS) was employed for gel aging. All reagents were sourced from Sigma-Aldrich.

The synthesis of the hydrophobic silica aerogels was performed following the guidelines from previous work [26]. The procedure began by diluting SS in distilled water to achieve a solution of 8 wt.% SiO_2_ (initial pH = 11). The sodium silicate solution was prepared by diluting 2.6 mL of SS in distilled water in a 50 mL beaker. TMES was added to the solution at a molar ratio of 1:1, corresponding to 2.4 mL of TMES, with constant stirring to obtain initial hydrophilization. Next, 2 mL of enzyme solution was added while stirring and HNO_3_ 0.5 M was added dropwise until a pH score of 9 was reached. The solutions were poured into cylindrical plastic molds to allow gelation (gel time ~ 10 min).

The resulting gels were washed six times in 48 h using distilled water and then aged in a solution of 80% vol. TEOS/isopropyl alcohol for 24 h at room temperature. After aging, the samples were subjected to a second hydrophilization process through surface derivatization by soaking in a solution of 20 vol.% TMES and ethanol for 24 h at room temperature. The samples were dried supercritically using CO_2_ as a supercritical fluid. Table 2 summarizes the processing conditions of the different aerogel samples [26].

For initial characterization of the samples, FT-IR analyses were performed using a Thermo Scientific Nicolet 300 (Waltham, MA, USA) spectrometer with an ATR accessory. The samples were crushed and spectra were obtained from 4000 to 650 cm^−1^ with 64 sweeps in absorbance mode. For TEM analysis, the aerogels were crushed, dispersed in ethanol, and ultrasonicated for 25 min. Afterward, a drop of the dispersion was placed in a Cu/C grid and left at room temperature for evaporation [26].

The aerogel samples were processed for contact angle measurements to obtain uniform pieces, ensuring consistent surface properties for accurate testing. The measurements were conducted using a ramé-hart goniometer. A droplet of distilled water was gently placed on the surface of each aerogel sample, and the contact angle was recorded immediately after the droplet made contact. The mean contact angle was then calculated for each sample.

The aerogel samples were degassed using a vacuum (<133.2 Pa) at 333.15 K for 24 h. The nitrogen adsorption experiments were conducted in a vacuum line, during cooling of the samples with liquid nitrogen (77.15 K). The specific total porosity (*V*_total_), microporosity (*V*_micro_), mesoporosity (*V*_meso_), and macroporosity (*V*_macro_) were quantified with the specific adsorption values at the relative pressures *p*/*p*_0_ = 0.05 (filled micropores), *p*/*p*_0_ = 0.40 (filled micropores and mesopores), and *p*/*p*_0_ = 0.98 (total porosity filled), according to [62].

Experiments involving hyperpolarized ^129^Xe NMR were conducted using a Bruker DRX-600 MHz spectrometer (Bruker Biospin, Rheinstetten, Germany) with a 5 mm TBI probe, operating at a Larmor frequency of approximately 166.00 MHz. The hyperpolarization process employed spin-exchange optical pumping (SEOP, Irvine, CA, USA) under continuous-flow conditions, facilitated by a custom-built polarizer, as detailed in previous studies [63].

A specific gas mixture was prepared, consisting of 1.1–1.4% xenon (natural isotopic distribution, Air Liquide, purity: 99.998%), 27.4–27.5% nitrogen (Air Liquide, purity: 99.999%), and 71.2–71.4% helium (Air Liquide, purity: 99.999%). The optical pumping cell was maintained at temperatures between 380 K and 425 K, utilizing metallic rubidium (Alpha Aesar, purity > 99%) as part of the system.

Prior to conducting the NMR measurements, the samples were activated for 12 to 14 h at 350 K. They were then placed in a 5 mm NMR tube equipped with a gas insertion system to facilitate continuous gas delivery. For data acquisition, one-dimensional spectra were generated through a total of 1024 scans, incorporating a recycle delay of 1s. Temperature calibration was achieved using the proton chemical shift of methanol as a standard. The spectra were processed using the MestReNova software (Mestrelab Research, Version 14.1.0).

The ^29^Si and ^13^C CP (cross-polarization)-MAS experiments were performed using a Bruker AVANCE-III 400 MHz WB NMR spectrometer (Rheinstetten, Germany) equipped with a 4 mm double-resonance MAS probe. In each experiment, for the maximal overall signal intensity, 4 mm zirconia MAS rotors were fully packed with around 15 mg of the aerogel sample. The readout temperature of 298 K was maintained, with a deviation of ±0.2 K by a temperature control unit. A MAS rate of 12,500 ± 3 Hz was controlled by a Bruker MAS unit. The optimized ^1^H, ^29^Si, and ^13^C, π/2 pulse lengths were 2.5, 4.8, and 3 µs, respectively. ^13^C and ^29^Si transverse magnetization created by ramped CP (70–100%) was transferred from ^1^H with an optimal contact time of 2 ms. For ^29^Si and ^13^C measurements, r.f. lock fields of 29.0 and 66.8 kHz were applied, fulfilling the Hartmann–Hahn condition. During the acquisition, a swept-frequency two-pulse phase modulation heteronuclear decoupling (SWf-TPPM) at a ^1^H r.f. field of 100 kHz was used for ^1^H decoupling. For the 1D ^29^Si and ^13^C CP-MAS spectra of the A0E24 sample, 77,401 scans for ^29^Si and 38,122 scans for ^13^C were accumulated, with a relaxation delay time of 2 s. ^29^Si and ^13^C chemical shifts were referenced to the signal of solid tetrakis (trimethylsilyl)silane at 3.6 ppm and to the COO^−^ signal of solid L-tyrosine·HCl at 172.1 ppm, respectively. The data were processed with Bruker Topspin 4.3.0 and further analyzed with MestReNova 15.0 (Mestrelab Research, Santiago de Compostella, Spain).

**Figure 7:**
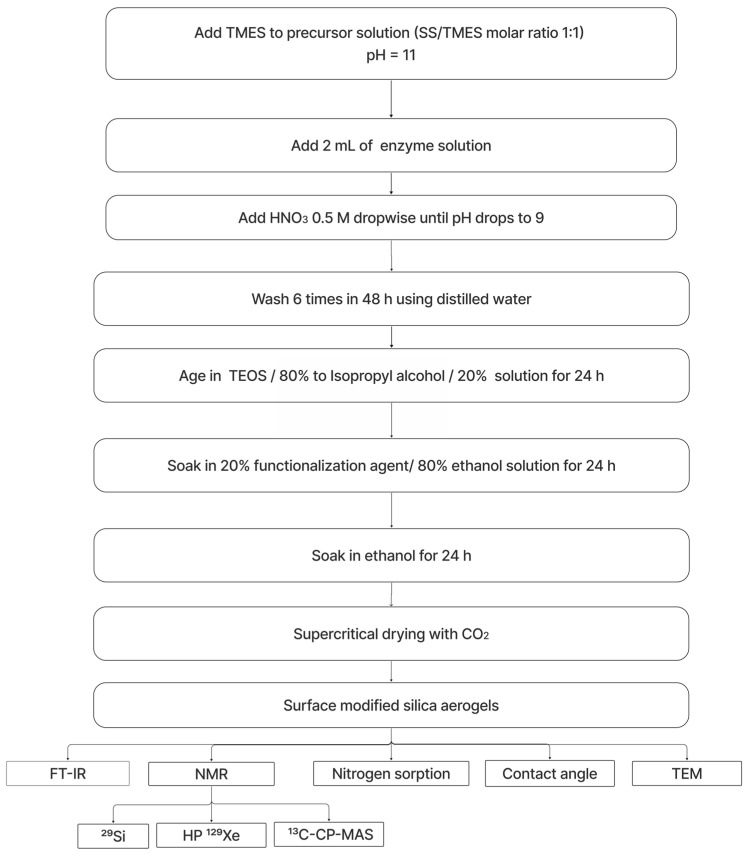
The procedure flowchart for the enzyme-encapsulated aerogel samples.

Figure 7 represents the flowchart of the process for enzyme-encapsulated modified silica aerogels. 

## Figures and Tables

**Table 1:** The physical properties of the synthesized samples.

Sample	BET Surface Area (m^2^ g^−1^)	Pore Volume (cm^3^ g^−1^)	Average Contact Angle (°)
A024	338	1.061 ± 0.007	128.56
A0E24	330	0.996 ± 0.005	148.37

**Table 2:** The synthesis parameters of the produced samples.

Sample	Functionalization Agent	Aging Time (h)	Enzyme Solution (mL)
A024	TMES 1:1	24	0
A0E24	TMES 1:1	24	2

## Data Availability

The original contributions presented in this study are included in the article. Further inquiries can be directed to the corresponding authors.
